# Pinpointing Protein Crystal Structures over a Broad Temperature Range Using Hydrophobic Protection

**DOI:** 10.1021/acsomega.5c13533

**Published:** 2026-05-22

**Authors:** Fernando de Sá Ribeiro, Luís Maurício T. R. Lima

**Affiliations:** Laboratório de Biotecnologia Farmacêutica (pbiotech), Faculdade de Farmácia, Universidade Federal do Rio de Janeiro, Rio de Janeiro 21941-902, Brazil

## Abstract

Protein interactions are dynamic processes that are influenced by chemical and physical variables. While variable temperatures play an important role in understanding biological processes, their use in protein crystallography has been limited because of the loss of diffraction power. Here, we report the selection of a cryoprotectant that enables X-ray diffraction data collection across a temperature range from cryogenic to room temperature. Although several hydrophobic materials effectively preserved samples at 100 K, only a few compounds allowed for data collection at 300 K. We identified hydrophobic greases suitable for both home-source and synchrotron applications, supporting fast and slow diffraction, with long exposure times at atomic resolution. Data collected between 100 and 300 K showed no significant effect of exposure time (ranging from 70 ms to 1 s) but revealed an exponential temperature dependence on the overall B-factor. These findings highlight the importance of hydrophobic grease in crystal protection against variables, such as temperature and dehydration, enabling optimized data collection across a wide temperature range.

## Graphical abstract

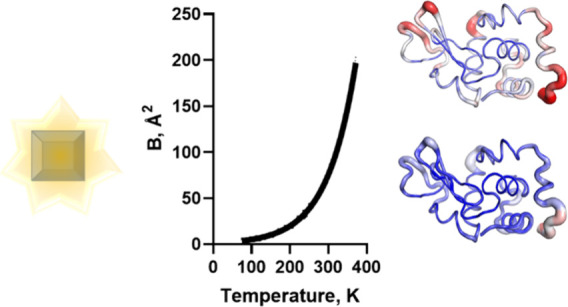

## 1 Introduction

Water is a noble molecule in life,^1^ and great breakthroughs in structural biology have come upon learning how to control its properties.

In protein crystallography, the key event opening the road for its development was made in the 1930s by Prof. John Desmond Bernal and Prof. Dorothy Crowfoot Hodgkin, with their landmark observation that crystals should be protected against dehydration to maintain order and diffraction.^2–4^

Another major development was the achievement of diffraction under subzero temperatures, denominated cryo-crystallography, paved by the demonstration by Prof. Gregory Petsko in 1975 that replacing or doping the mother liquor with organic compounds resulted in water vitrification, allowing protein single crystal X-ray diffraction at subzero temperatures^5^ in the absence of ice, and data collection across a broad temperature range (220–300 K).^6^ Later in 1992, Prof. Petsko and his group further used this solvent replacement approach to demonstrate that an apparent biphasic transition occurs in RNase crystal diffraction over a temperature transition from cryo (98 K) to high (320 K) temperature,^7,8^ using different cosolvent (2-methyl-2,4-pentanodiol; methanol) and crystal mounting (capillary; glass fiber) strategies at each temperature. They explained the apparent biphasic transition observed in the Debye–Waller factor as a function of temperature seen in their work^6–8^ as being dominated by a harmonic motion at higher temperatures, progressively decreasing and dominated by the harmonic vibration of individual atoms at temperatures below 200 K.

The B-factor, or temperature factor, is related to the mean-square atomic displacement (*X* ^2^) by the Debye–Waller function:

$$B=8\pi^{2}X^{2}$$ 1

The *X* ^2^ comprises contributions from atomic fluctuation (*X* ^2^_A_), conformational motions (*X* ^2^_C_), rigid body vibration (*X* ^2^_RB_), and crystal lattice disorders (*X* ^2^_LT_),^9^ which can be dissected by exploring physical variables affecting the *B*-factor. However, while the crystal structure is highly reproducible,^10,11^ the *B*-factor varies largely between crystals, instruments, and cosolvents, limiting the use of multiple single crystals in the investigation of effects of variables in the *B*-factor or in serial crystallography.^12–14^

The reproducibility of the *B*-factor was finally achieved by mathematical normalization:^12,15^

$$B_{\mathrm{norm}}=B_{\mathrm{obs}}/B_{\mathrm{avg}}$$ 2

where *B*_norm_ is the normalized *B*-factor, *B*_avg_ is the average *B*-factor, and *B*_obs_ is the observed raw *B*-factor. While normalization allows several analyses, including correlations between *B*-factor and structural changes, and can be easily implemented in data processing pipelines in structural biology methods such as single-crystal and serial-crystal X-ray diffraction, cryo-EM,^16,17^ and others^18,19^ (exporting PDB with a normalized *B*-factor), it does not allow interpreting the absolute Debye–Waller factor.

The experimental reproducibility in *B*-factor could be achieved by crystal stabilization using hydrocarbon grease,^13,14^ allowing demonstration of the dominance of whole-system rigid-body motion on the temperature-dependence *B*-factor change, well described by a single-exponential dependence of the *B*-factor as a function of temperature for all atoms of the system (protein/nonprotein) with a thermal constant “*k*” of about 0.004 K^–1^ as follows:

$$B_{\mathrm{obs}}=B_{0}\times e^{\left(k\times T\right)}$$ 3

where *B*_0_ is the *B*-factor extrapolated to zero Kelvin from regression (about 5 Å^2^), *k* is the thermal constant, and *T* is the data collection temperature (in Kelvin). After the advances obtained with cryo-crystallography, a recent revival of the room temperature data collection returned to the spotlight,^20–22^ motivated by serial crystallography. Alternative nondehydration techniques have been pursued, such as tubbing (capillaries or other polymer devices),^23^ glues,^7,24^ silicone,^25^ lipids,^26^ oils,^27–30^ mineral oil-based grease,^31^ vegetable,^32^ and animal fat.^33,34^ A recent work on the X-ray diffraction at high resolution (1.40–1.68 Å) of tetragonal Proteinase-K from room temperature (293 K) to extreme high temperatures (363 K, 90 °C), conducted with crystals embedded into Paratone-N oil as initially proposed by Hope in 1988,^27^ suggested a progressive increase in the Wilson B-factor (from 23 to 34 Å^2^) as a function of temperature.^35^ Unfortunately, this work did not address temperatures below 20 °C.

The comparative use of these crystal stabilization approaches for broad-range temperature measurements without changes in crystal composition has not been tested thoroughly, and there is a large gap between cryogenic and room temperatures in the literature and the PDB database that still needs to be explored (Figure 1).

**Figure 1:**
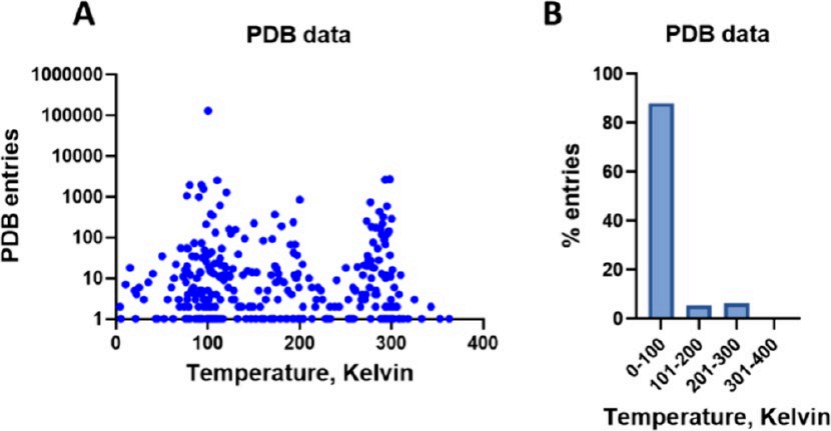
Distribution of reported X-ray diffraction temperature. Data were retrieved from the RCSB (accessed May, 2025). (A) All structures deposited in the PDB by temperature (notice log scale). (B) All structures deposited in the PDB by temperature range (100 K interval).

There are still several limitations, such as the study of continuous temperature dependence over a broad temperature range, from cryo to above room temperature, allowing exploration of the temperature effect on conformational changes, ligand interaction, and raw *B*-factor (not normalized), as well as conducting measurements at long exposure in a home source or synchrotron without major radiation damage.

In this study, we explore a large set of potential protective agents for data collection at cryogenic and ambient conditions and explore the protection against high radiation flux over a broad temperature range.

## 2 Materials and Methods

### 2.1 Materials

Hen egg white lysozyme (HEWL, UniProt ID P00698) was obtained from Fluka (Cat #62971; Lot #BCG4805V) and kept at −20 °C until use. Apiezon N and Apiezon T were obtained from Sigma-Aldrich. Vaseline (petroleum jelly; Vasenol), mineral oil (Farmax), lard (Sadia), and olive oil (De Cecco) were purchased from local stores. Silicone oil (Dow Corning 360 Medical Fluid, 350 CST) was a kind donation from Hygeia Biotech S/A. We did not test Paratone-N oil because we lacked access to it. All other reagents were of analytical grade.

### 2.2 Methods

#### 2.2.1 Protein Crystallography

Crystals of tetragonal (*P*4_3_2_1_2) HEWL were obtained by vapor diffusion, sitting-drop technique, using Corning 3552 plates with 1 μL protein solution (HEWL 50 mg/mL in water), and 1 μL precipitant composed by 100 mM sodium acetate pH 4.6, 1.7 M NaCl, with (synchrotron data collection) or without (home source data collection) 25% ethylene glycol (for faster crystallization for synchrotron measurements), equilibrated against 80 μL of precipitant solution at 20 °C ± 2 °C.^36^ Diffraction-quality crystals were used after 2 days. Crystals were manually picked using a 20 μm nylon CryoLoop (Hampton Research), soaked in the crystallization solution supplemented or not (in case of having ethylene glycol) with glycerol (for 30% v/v final concentration), followed by immersion into the oil cryoprotectant. The crystals were mounted onto the goniometer with N_2g_ stream set at the desired temperature for data collection, and a complete data set was collected as depicted below. For each data set, a new crystal was used.

#### 2.2.2 Data Collection and Processing

Two workflows were used in the X-ray diffraction and data collection.

##### 2.2.2.1 Home Source

Crystal diffraction was performed using CuKα radiation from a D8-Venture diffractometer (Bruker AXS Inc.; installed in the CENABIO-UFRJ) operating at 50 kV and 1.1 mA from a 30 W air-cooled |μS microfocus source (Incoatec), and images were recorded on a Photon II detector (Bruker). Temperature was controlled with a N_2g_ stream (flow of 1.2 L/h) set to the desired temperature, using a CryoStream 800 (Oxford Cryogenics). All data sets were collected with frames of 1 min exposure/0.5 ° oscillation, reaching at least 99% completeness and 1.5 Å resolution (for Friedel pairs treated as equals). All data collection, indexing, integration, and scaling were performed by *Proteum3* (Bruker AXS Inc.) and further analyzed with *Truncate—*C.C.P.4.^37^ The data range was from 10.66 to 1.50 Å.

##### 2.2.2.2 Synchrotron

Data were obtained by diffraction in the Manaca beamline from Sirius synchrotron (CNPEM, Campinas, SP, Brazil) with radiation at 16 keV (0.7749 Å) and collected with Pilatus 2 M (Dectris) detector set at 112 mm from crystal for all data sets, allowing a maximum resolution of 0.947 Å according to the data acquisition software MXCuBE (https://www.mxcube.org/). The temperature was controlled by a N_2g_ stream at 8 L/min using CryoStream 700 (Oxford Cryogenics). All data sets were collected with 0.5° oscillation, in a total of 180°, resulting in 360 images, processed with XDS implemented in MNCAutoproc^38,39^ written by Dr. Andrey Nascimento (Manaca beamline, Sirius, CNPEM, Campinas, SP, Brazil), based on the original Autoproc,^40^ set to resolution at CC_1/2_ = 50%. Beam attenuation was changed inversely proportional to exposure time per frame (70 ms, 1 s, 5 s, 30 s) in order to decrease flux (photons/s), resulting in a close total photon count per frame.

#### 2.2.3 Data Processing

All data were further processed with C.C.P.4. v7.0.071.^39^ Structures were solved by molecular replacement with rigid body refinement using RefMac v5.8.0238 ^41,42^ with PDB ID 3A8Z,^43^ followed by restrained refinement in *Refmac*.^41^ Further real-space modeling was performed with *C.O.O.T.* v0.8.9.2 ^44^ and an additional restrained refinement with *Refmac*, always in default mode to avoid bias.

Details of the crystal parameters, data collection, and refinement statistics are compiled in Table S1 (separated spreadsheet) of the Supporting Information. All molecular representations were created using PyMOL v2.0 (The PyMOL Molecular Graphics System). The final atomic coordinates have been submitted to the Protein Data Bank (PDB), with accession codes provided in the Supporting Information.

#### 2.2.4 Data Analysis

Graphics were generated with GraphPad Prism v. 10.1.2 for Windows (GraphPad Software, San Diego, California USA).

## 3 Results

### 3.1 Screening of Crystal Stabilizing Agents

We have tested embedding lysozyme crystals into varying compounds and evaluated the stability for X-ray data diffraction at cryogenic conditions (100 K) and room temperature. At 100 K, all tested compounds were satisfactory at keeping data diffraction at 1.5 Å, although resulting in dissimilar data diffraction quality as judged by the data collection parameters (Table 1). However, we observed a loss of diffraction power at room temperature for most stabilizing agents tested, except for the hydrocarbon greases Vaseline and Apiezon (Table 1).

**Table 1:** Analysis of Data Collection Parameters Using Varying Protective Agents^a^

	100 K					300 K				
	**a = b**	**c**	***W*** **_Bf_**	**^low^** ***I*/σ**	^**high**^ ***I*/σ**	**a = b**	**c**	***W*** **_Bf_**	^**low**^ ***I*/σ**	^**high**^ ***I*/σ**
Apiezon N	78.24	37.34	11.0	109.89	9.55	79.23	38.10	14.5	35.33	2.65
	±0.12	±0.05	±1.0	±6.53	±1.70	±0.06	±0.01	±1.1	±27.02	±0.76
Apiezon T	78.13	37.15	11.2	110.68	7.22	79.00	38.10	14.1	106.65	3.34
	±0.20	±0.08	±1.6	±9.69	±3.24	±0.09	±0.10	±3.4	±31.52	±0.42
glycerol	78.23	37.03	13.3	145.33	6.69	N/A				
	±0.30	±0.05	±1.2	±30.35	±3.20					
lard	78.38	37.26	11.0	101.98	9.67	N/A				
	±0.09	±0.08	±0.9	±5.85	±0.37					
mineral oil	78.55	37.16	10.7	190.47	14.61	N/A				
	±0.12	±0.05	±0.1	±21.64	±1.35					
No Cryo	77.89	37.27	13.4	101.62	3.86	N/A				
	±0.27	±0.06	±0.8	±15.25	±0.57					
olive oil	78.15	37.16	10.8	79.92	6.84	N/A				
	±0.19	±0.14	±0.7	±7.82	±0.85					
PEG 400	77.23	37.42	13.0	158.00	8.42	N/A				
	±0.12	±0.06	±1.0	±20.41	±2.56					
PEG 6000	78.53	37.16	11.2	159.96	9.18	N/A				
	±0.09	±0.01	±0.4	±11.95	±1.58					
silicone oil	78.48	37.13	11.3	184.69	10.51	N/A				
	±0.03	±0.03	±0.5	±24.83	±2.07					
vaseline	78.08	37.30	12.45	99.60	6.65	79.08	38.26	14.2	92.32	3.12
	±0.49	±0.07	±0.7	±6.40	0.80	±0.13	0.17	±1.6	±29.32	±0.22

a a, b, and c are cell parameters (Å). ^*W*^Bf is the Wilson *B*-factor. ^low^ *I*/σ_Avg_ is low-resolution intensity/sigma. ^high^ *I*/σ is high-resolution intensity/sigma. N/A—not available, due to loss of diffraction power. Lysozyme crystals (*n* = 3 per condition; average and standard deviation) were embedded into candidate stabilizing agents and subjected to full X-ray data collection at a target resolution of 1.5 Å.

Although all stabilizing agents allowed full single crystal X-ray data diffraction and structure solving at the same target resolution (1.5 Å), they impacted the data quality (Table 1 and Figure 2). A positive correlation between *I*/σ at high resolution and low resolution was observed (*p* = 0.022), with a negative correlation between *I*/σ at high resolution and the Wilson *B*-factor (*p* = 0.030) (Figure 2). Although the higher intensity with a lower Wilson *B*-factor was obtained with mineral oil, this tested agent did not allow data collection at 300 K. The other better agents under these criteria for data collection at 100 K were silicone oil and Apiezon, although silicone oil also resulted in low of diffraction power at 300 K. The hydrocarbon grease Apiezon N is recommended for a working range of 4–303 K (−269 to 30 °C), while the Apiezon T is recommended for temperature ranging from 283 to 393 K (10–120 °C).

**Figure 2:**
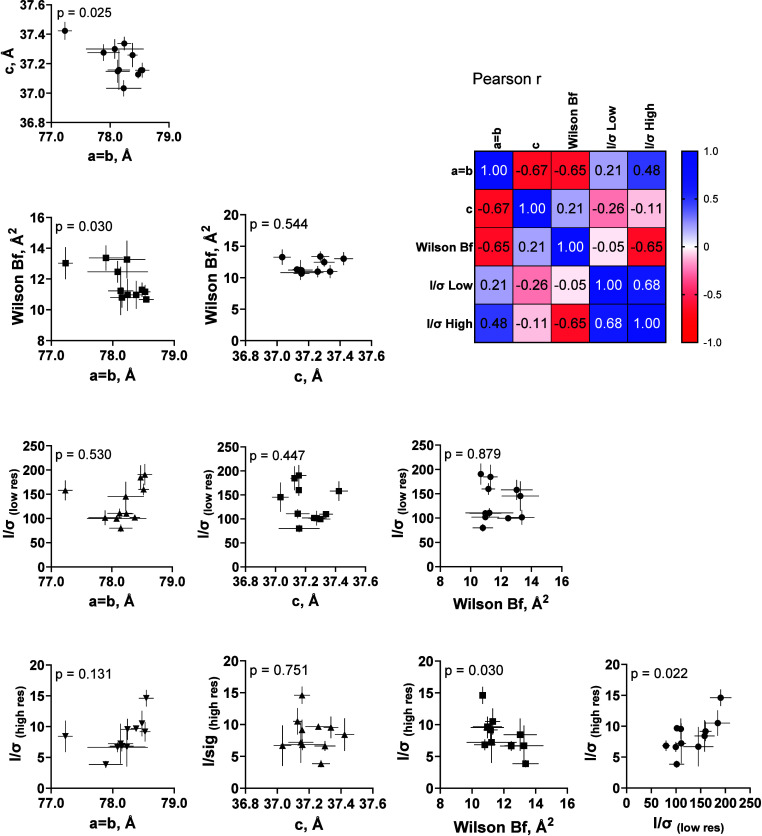
Correlation analysis between data with varying stabilizing agents. Pearson correlation r and p-value were obtained for data obtained from the full data set collected from X-ray single-crystal diffraction at 100 K. Symbols and bars represent average and standard deviation (*n* = 3), respectively.

The structural findings showed a great similarity between all models in both cases (100 K and room temperature) (Figure 3). The *B*-factor analysis of the structures reveals an overall increase from data collected at 100 and 300 K for all conditions, which becomes similar in distribution over the polypeptide chain upon normalization (eq 2), indicating no major distinction between them (Figure 4). Since we aimed to collect data under varying temperatures as a continuous variable, from cryo to room temperature, we chose the Apiezon N for further investigations.

**Figure 3:**
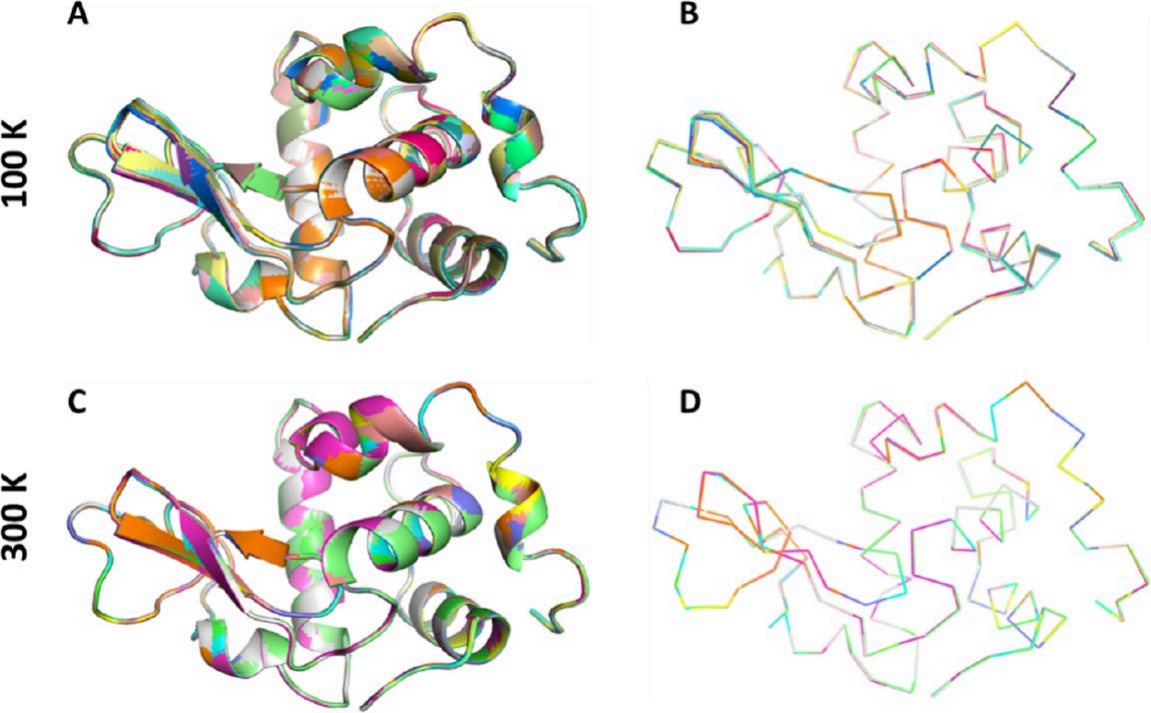
Alignment of crystal structures. Structures solved using home-source X-ray and varying stabilizing agents (Table 1; *n* = 3/compound) were superposed using PyMOL. Each color represents one structure. (A) 100 K, cartoon representation. (B) 100 K, backbone representation. (C) 300 K, cartoon representation. (D) 300 K, backbone representation.

**Figure 4:**
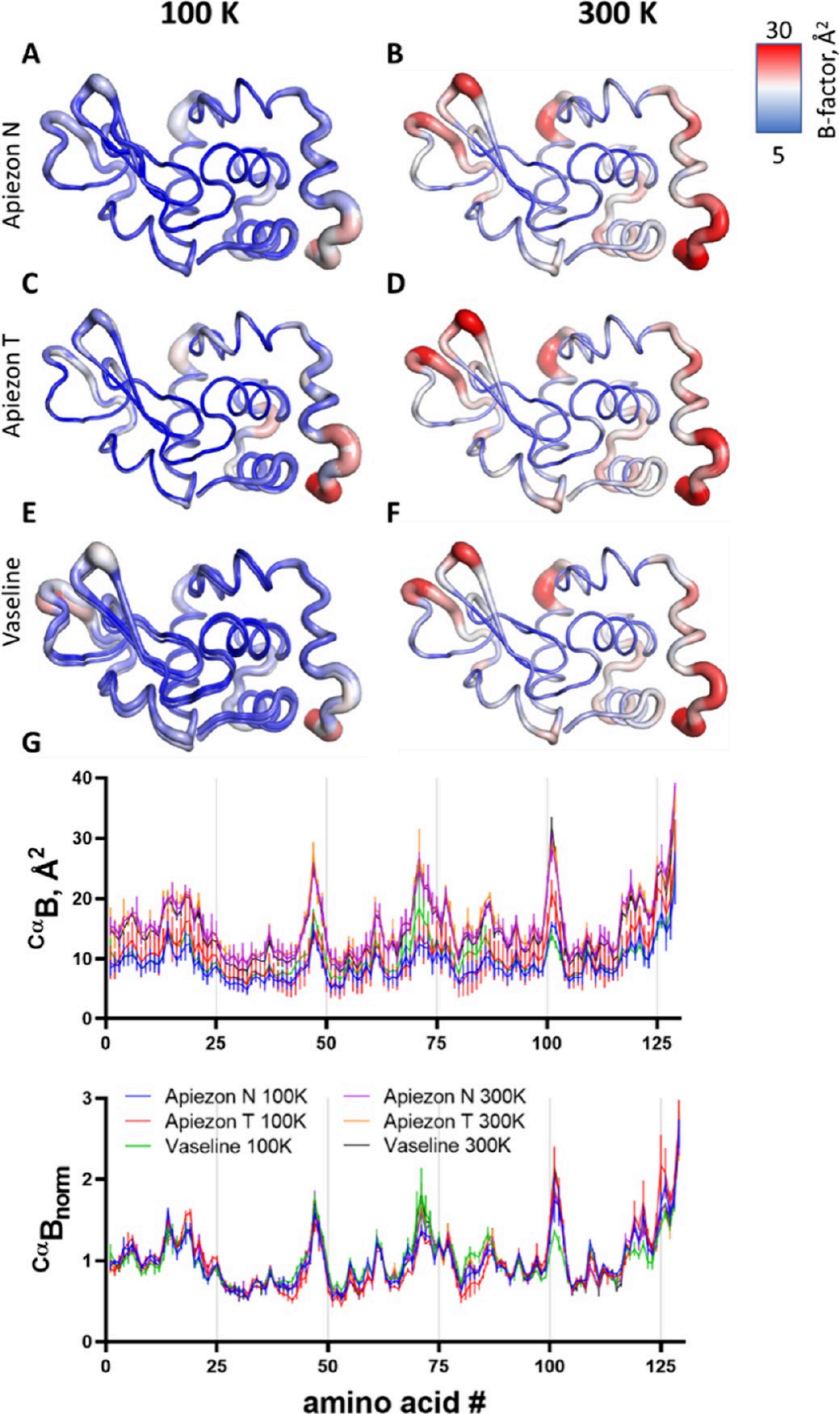
*B*-factor distribution in crystal structures. Structures solved from single-crystal X-ray diffraction data (*n* = 3/group) using varying stabilizing agents are represented by the *B*-factor scale according to the presented color scale. (A) Apiezon N (100 K); (B) Apiezon N (300 K); (C) Apiezon T (100 K); (D) Apiezon T (300 K); (E) Vaseline (100 K); (F) Vaseline (300 K). (G) Raw and the normalized *B*-factors for each structure (average and standard deviation; *n* = 3/condition) are presented.

### 3.2 Crystal Protection Against Short- and Long-Exposure Synchrotron Radiation

We further tested the crystal quality stability against short and prolonged data collection using synchrotron radiation. Crystal diffraction data collection under 5 and 30 s exposure per frame, attenuated proportionally for similar photon dose, did not affect the resolution, *B*-factor, or cell parameters substantially, despite an increase in *I*/σ in the low resolution without benefits in resolution (Table 2). Thus, the use of hydrocarbon grease could benefit longer exposures for low-diffracting crystals.

**Table 2:** Exposure Time Dependence on X-ray Diffraction Data Quality by Protein Crystals^a^

**exp. time, s**	**transmission, %**	**flux** (ph/s)	**dose/frame**	**res., Å** (CC_1/2_ **= 0.5)**	***I*/sig low**	**Wilson Bf, Å** ^**2**^	***a* = *b*, Å**	***c*, Å**
0.07	100	3.56 × 10^11^	2.49 × 10^10^	1.02	51.39	9.1	78.96	36.75
1	10	3.48 × 10^10^	3.48 × 10^10^	0.99	63.05	9.2	79.02	36.77
5	2	6.08 × 10^09^	3.04 × 10^10^	0.99	71.22	8.9	79.00	36.80
30	0.3	8.91 × 10^08^	2.67 × 10^10^	1.00	74.89	8.7	79.03	36.80

a The full dataset was collected from HEWL crystals embedded into Apiezon N with varying exposure time at 100 K, compensated for transmission for close dose exposure per frame.

### 3.3 Thermal Dependence of Crystal Quality Upon Synchrotron Radiation X-ray Diffraction Data Collection

Tetragonal lysozyme crystals embedded into the hydrocarbon grease were subjected to full data set X-ray diffraction from 100 to 300 K in 25 K intervals, with varying exposure time (70 and 1000 ms) and attenuation resulting in similar dose/frame. Data from integration revealed minor variations in cell dimensions (*a* = *b* vs *c*; Figure 5A), which were linearly correlated (Pearson *r* = 0.82, 95% CI = 0.65–0.92, *p* < 0.0001) (Figure 5B).

**Figure 5:**
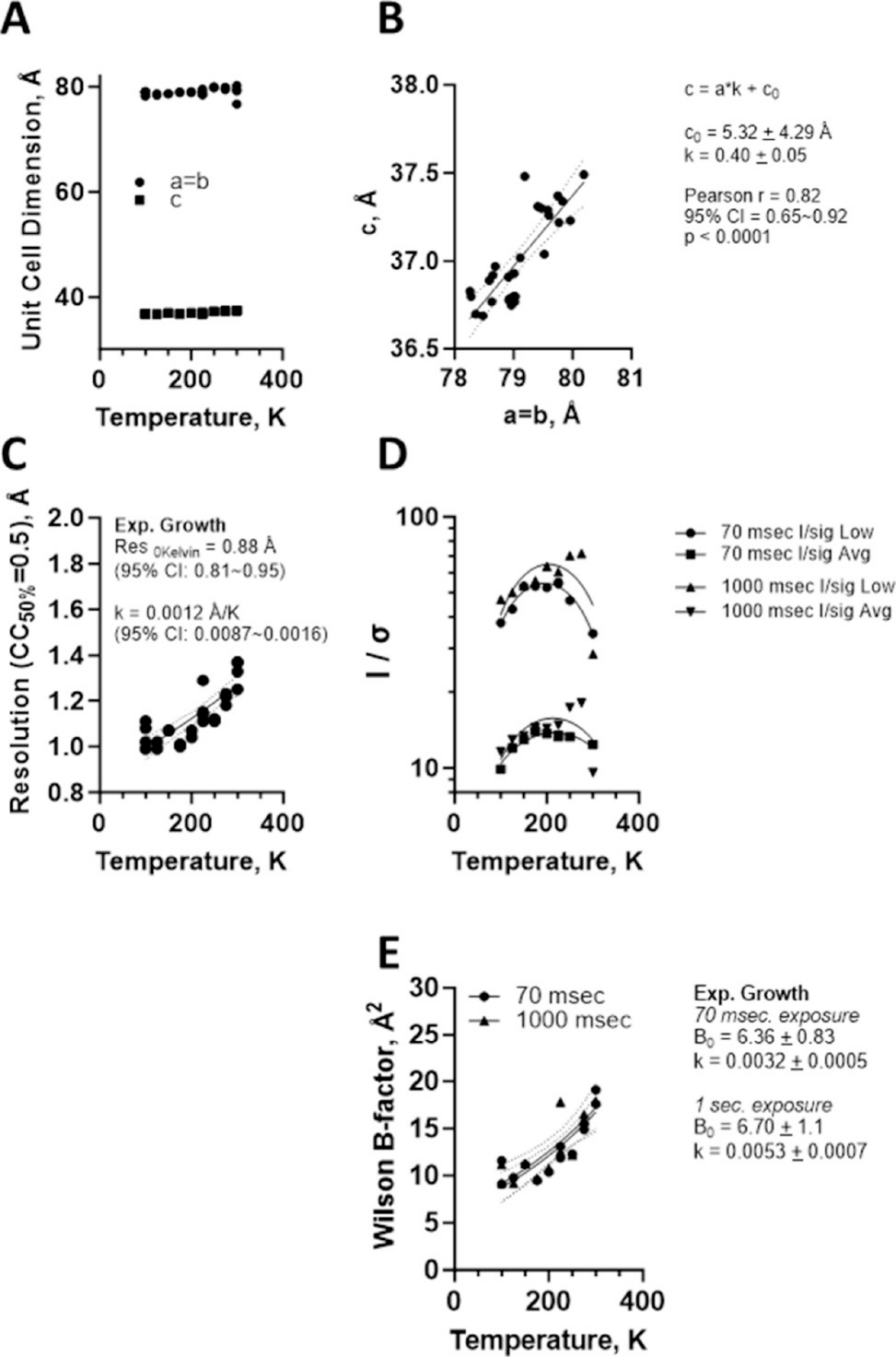
Temperature-dependent effect on X-ray diffraction crystal properties. Crystals embedded with Apiezon N were diffracted using synchrotron radiation, and data quality was evaluated against temperature for (A) cell parameters, (B) correlation between cell parameters, (C) resolution threshold, (D) *I*/sigma, and (E) Wilson *B*-factor.

The resolution limit, automatically defined by CC_1/2_ = 0.5 as a criterion, showed a small but progressive decrease from about 1.0 Å at 100 K to about 1.3 Å at 300 K (Figure 5C).

There occurred a biphasic dependence of the reflection intensity as a function of increasing temperature (Figure 5D), increasing from 100 K to about 200 K, followed by a decrease up to 300 K.

The Wilson *B*-factor showed an exponential increase as a function of temperature, with overall extrapolated zero-point *B*-factor ^Wilson^Bf_0_ = 6.27 ± 0.56 Å^2^ (*B*_0_ = 6.36 + 0.83 for 70 ms, and *B*_0_ = 6.70 ± 1.1 for 1 s exposure) and exponential factor *k* = 0.0034 ± 0.0004 Å^2^/K (*k* = 0.0032 ± 0.0005 for 70 ms, and *k* = 0.0053 ± 0.0007 for 1 s exposure) (Figure 5E).

These data suggest the ability of the hydrocarbon grease to support data diffraction continuously over a broad range of temperature using both home source and synchrotron radiation at both short and long X-ray exposure. However, further investigations with other proteins stabilized with hydrophobic greases are required to confirm the extension of such an observation to other systems and the transition observed at temperatures close to 200 K.

## 4 Discussion

Proteins are highly dynamic and can populate a vast conformational space. Characterizing these polymorphs requires chemical and physical variables associated with spectroscopic and other state-of-the-art techniques, such as NMR, cryoEM, and crystallography. While NMR and cryoEM data collection are performed in orthogonal temperature conditions (above zero Celsius for NMR, and around 77 K for cryoEM, liquid nitrogen temperature, although reaching high *B*-factors close to or above 100 Å^2^, Figure S3), crystallography could benefit from near-zero temperature^45^ up to 90 °C.^46^ However, a significant gap in crystallography persists between cryogenic (∼100 K) and room-temperature conditions—a “cryogenic valley of death”—mainly due to the water phase transition near 200 K. In this work, we evaluated various readily available compounds as stabilizing agents for single-crystal X-ray diffraction data collection across a temperature range from cryogenic to room temperature. Among these, we identify the effectiveness of vaseline and Apiezon—hydrocarbon greases known for their broad temperature stability, low impurity levels, and similar, low background scattering in the high resolution range (<3 Å) (Figures S1 and S2).^47^ Apiezon N, in particular, offered advantages due to its viscosity, which promotes crystal adherence and prevents dehydration during prolonged exposure (hours). While the natural greases worked at 100 K, they could not provide the same durable barrier at 300 K that the hydrocarbon greases offered, perhaps due to temperature- and radiation-mediated oxidation. Apiezon greases may have some limitations. Their transparency is lower than that of oils, which may restrict the size of crystals suitable for diffraction. Additionally, while Apiezon is effective for single-crystal diffraction over the tested temperature range, its high viscosity could pose challenges for serial crystallography and require further optimization. In this study, we found that animal and vegetable greases were unsuitable for single-crystal X-ray diffraction at room temperature (Table 1), despite prior demonstrations of their applicability in serial crystallography.^32–34^ We were unable to test these compounds for serial crystallography due to a lack of access to the technique.

The advent of cryocrystallography enabled long X-ray exposures with high-resolution diffraction, particularly under high-intensity beamlines.^48^ Hope^27^ proposed a linear relationship between temperature and the *B*-factor for crystals embedded in Paratone-N oil:

$$B_{\mathrm{obs}}=B_{0}+k_{1}T$$ 4

where *B*_obs_ is the *B*-factor at temperature *T*, *B*_0_ is the ‘zero-point’ *B*-factor (at zero Kelvin), and *k* is a linear proportionality constant. Later, Petsko and colleagues^7^ suggested a biphasic model, with distinct linear relationships below and above 200 K:

$$B_{\mathrm{obs}}=B_{0}+k_{1}T+k_{2}T$$ 5

In their review on protein dynamics, Ringe and Petsko^9^ noted that while mean-square displacement *X* ^2^ could be refined with precision, determining their absolute accuracy remained challenging. Reproducible absolute *B*-factors were only achieved through stabilization with hydrophobic grease.^13,14^ Ringe and Petsko further hypothesized that below 100 K, the *B*-factor would become temperature-independent and uniform across all atoms, yielding an extrapolated zero point *B*-factor of 0.4 Å^2^.^9^

Contrary to this biphasic model, our data—spanning cryogenic to room temperature (100 to 325 K) at 1.5 Å resolution—demonstrate that the temperature dependence of the *B*-factor follows a single-exponential (eq 3). Using this model, we derived a thermal dependence constant (“*k*”) of about 0.0045 ± 0.0003 K^–1^ and a zero-point *B*-factor *B*_0_ of about 5.7 ± 0.5 Å^2^ from home-source diffractometer data (∼3 h of data collection time). Synchrotron data (70 ms to 1 s exposure/image at 1.0–1.3 Å resolution) yielded *k* = 0.0034 ± 0.0004 K^–1^ and *B*_0_ = 6.3 ± 0.6 Å^2^. Notably, hydrocarbon grease compatibility was further supported by consistent results across fast (70 ms) and long (30 s) synchrotron exposure (Table 2). These regression parameters, thermal constant *k* and the *B*_0_, are in good agreement with data recently reported independently for an unrelated protein obtained with Paratone oil (although with a gap between 200 and 300 K).^30^ Further broad-range multitemperature studies with other proteins are required to understand the limitations and validity of the present findings.

It is important to notice that this is a methodological study with findings demonstrated using lysozyme. To validate the thermal dependence of the crystallographic *B*-factor and the conformational effect across a broad temperature range (from cryo to above room temperature) in proteins, further studies should include proteins in the current repertoire.^30,55^ If possible, studies should include extremely low temperatures (below 100 K), which will require special setups such as helium cooling, in beamlines such as the EMA (Extreme condition Methods of Analysis) Beamline at Sirius, Brazil. Confirming the universality of the proportionality constant (“*k*”) and zero-point *B*-factor *B*_0_—both from raw data (Wilson *B*-factor) and refined atomic models—would clarify molecular motion in the crystalline state. Such verification could also drive broader advances in structural biology. In addition, advances in cryogenic data collection may revive subzero temperature-dependent structural biology, offering insights inaccessible to techniques such as NMR and cryo-electron-microscopy (cryoEM).

## 5 Conclusions

The main purpose of this work is to search for, and perhaps find, a stabilizing agent that would allow the use of temperature as a continuous variable in macromolecular crystallography, from cryogenic to above-room-temperature conditions. Hydrophobic stabilizers (e.g., Apiezon grease) were found to mitigate lysozyme crystal instability, enabling temperature-dependent studies across cryo-to-room-temperature regimes, an approach that can provide access to raw, nonnormalized B-factors and use them for further studies aiming to solve harmonic/anharmonic vibrational modes and their coupling to conformational dynamics. Further studies with other proteins and extremely low temperatures will assist in understanding the general validity of the vibrational properties of molecules in the crystal phase.

## Supplementary Material





## Data Availability

The experimental information and data supporting the findings of this study are available within the paper and the indicated data repository, under PDB ID listed in Table S1 for data collected at 100 K using Apiezon N (9P0Y, 9P0Z, 9P10), Apiezon T (9P56, 9P57, 9P58), lard (9P5B, 9P5D, 9P1W), glycerol (9P59, 9P5A, 9P5E), mineral oil (9P5F, 9P5G, 9P5H), no cryo (9P5J, 9P5K, 9P5L), olive oil (9P5M, 9P5N, 9P5O), PEG 400 (9P5P, 9P5Q, 9P5R), PEG 6,000 (9P5S, 9P5T, 9P5U), silicone oil (9P5 V, 9P5W, 9P5X), vaseline (9P5Y, 9P5Z, 9P60), and at 300 K using Apiezon N (9P61, 9P62, 9P63), Apiezon T (9P64, 9P65, 9P66) or vaseline (9P67, 9P68, 9P69). Diffraction images are available in the https://proteindiffraction.org/ using the same PDB code. Further information is available from the corresponding authors upon reasonable request.

## Notes

*I*/σ distribution by resolution, background XRD patterns of selected greases, and distribution of *B*-factors for cryoEM data (PDF)
Crystallographic data (XLSX)

## Notes

Hen egg white lysozyme (UNIPROT ID P00698).

## Abbreviations

HEWL: hen egg white lysozyme
