# Supplementary material for: Pinpointing Protein Crystal Structures over a Broad Temperature Range Using Hydrophobic Protection
Source: ACS Omega. 2026 May 22;11(22):32336–44. doi: 10.1021/acsomega.5c13533 (PMC13261484; doi:10.1021/acsomega.5c13533)
Supplement: Supplementary file 1 [file ao5c13533_si_001.pdf]

Supporting Information

Pinpointing protein crystal structures over broad temperature range using hydrophobic protection

Fernando de Sá Ribeiro[1*] and Luís Maurício T. R. Lima[1*]

[1] Laboratório de Biotecnologia Farmacêutica (pbiotech), Faculdade de Farmácia, Universidade Federal do Rio de Janeiro, Rio de Janeiro, RJ, 21941-902, Brazil.

*To whom correspondence should be addressed

AUTHOR LIST
Fernando de Sá Ribeiro – FernandoRibeiroBiomed@gmail.com
Luís Maurício T. R. Lima – LuisMauricioLima@gmail.com , Mauricio@farmacia.ufrj.br

Table S1. Crystallographic data. Crystals collected at home-source and synchrotron were processed, and a summary of the statistical data is presented in the corresponding spreadsheet associated with this work, including Temperature, protectant agent, PDB ID, cell parameters, Wilson B-factor, average B-factor, I/σLow, I/σHigh, $CC_{1/2}$, Completeness, among others. – please refer to file **ao5c13533_si_002.xlsx**

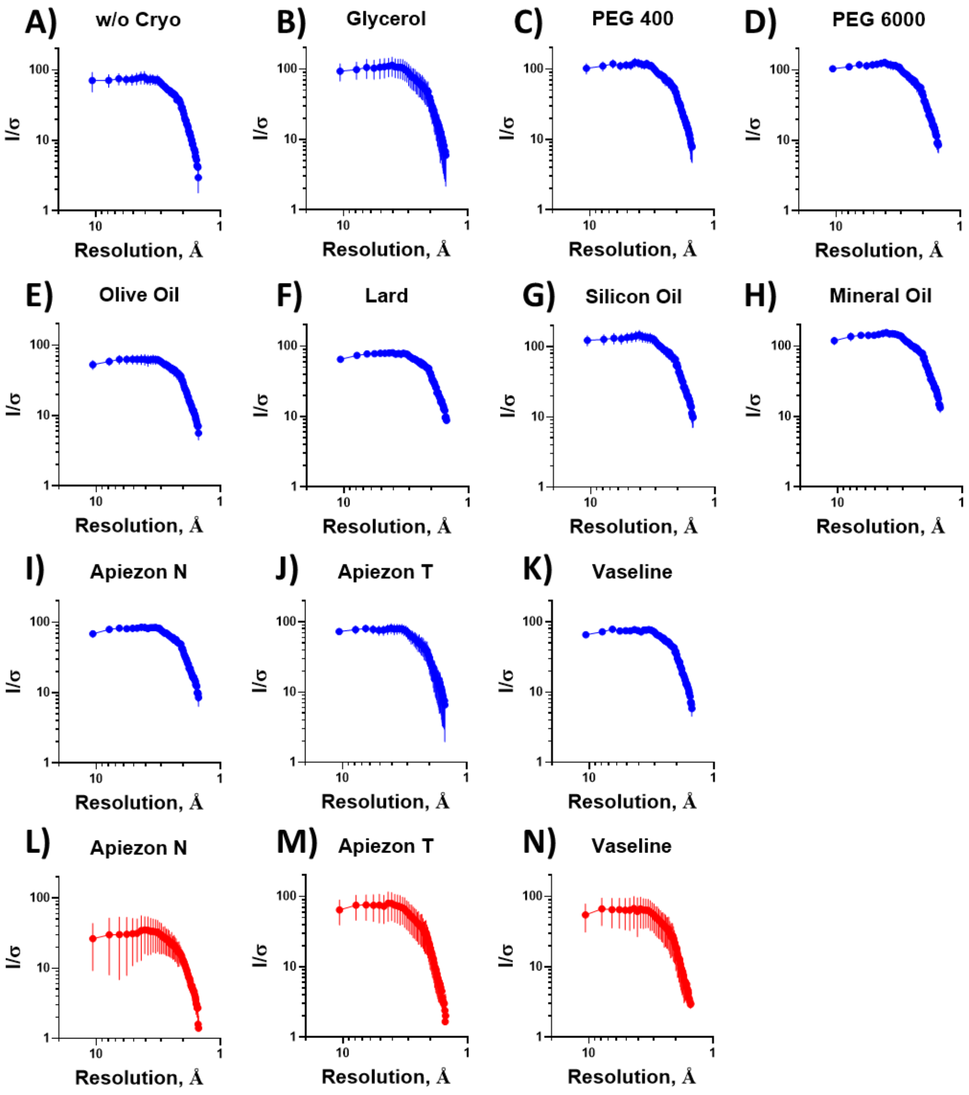

Figure S1. I/σ distribution by resolution. Crystals were diffracted using home-source CuKα radiation at 1.5 Å resolution from D8 Venture diffractometer (Bruker AXS Inc.), integrated using Proteum*3* (Bruker AXS Inc.) and scaled using Aimless (CCP4). The Wilson plot is presented for crystals (n=3 per condition) collected at **100 K** (A – K) and **300 K** (L – N). Details in the Experimental Section of the corresponding manuscript.

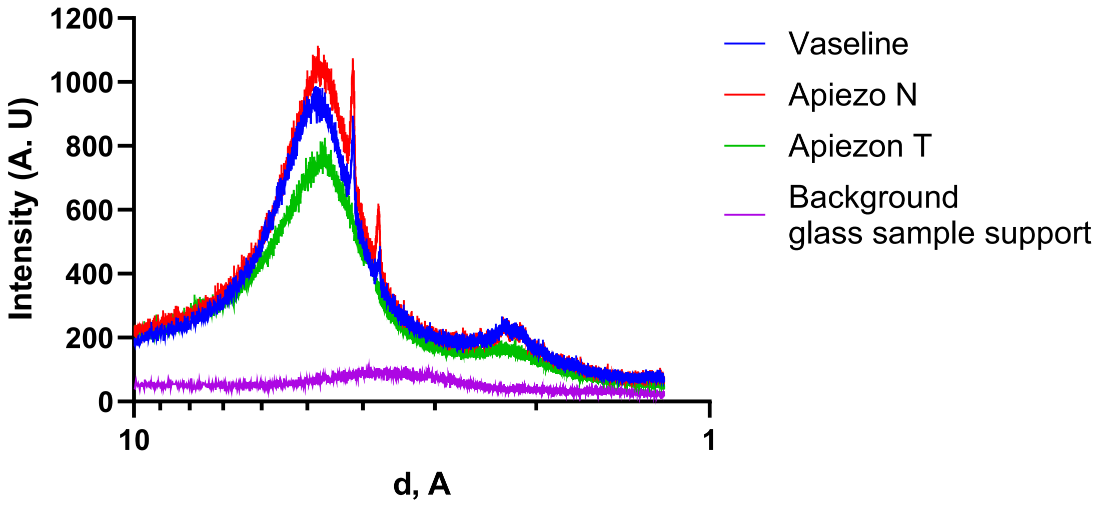

Figure S2. Background XRD patterns of selected greases. The evaluation of the background contribution of the grease alone (Vaseline, Apiezon N, and Apiezon T) was performed in the SuperNova (not shown; Agilent; Rigaku; INMETRO; data acquisition and processing performed using CrysAlisPro) with CuKα (1.542 Å) x-ray source operating at 50 kV and 50 mA, with the material supported by 10 µm nylon loop (Hampton Research), and continuous scanning in phi for 20° at 1°/sec, and confirmed in triplicate in the XRD-6100 diffractometer (above; Shimadzu; Fac. Farmacia, UFRJ; data collection and processing performed with Spoll ver. 7.00, Shimadzu Corp) with CuKα (1.542 Å) x-ray source operating at 40 kV and 30 mA, by depositing a uniform layer of the material onto glass support, and continuous scanning from 2° to 80° w in 0.02° step, with scanning at 2° / min, with divergence and scattering slits set at 1.000°, receiving slit 0.300 mm.

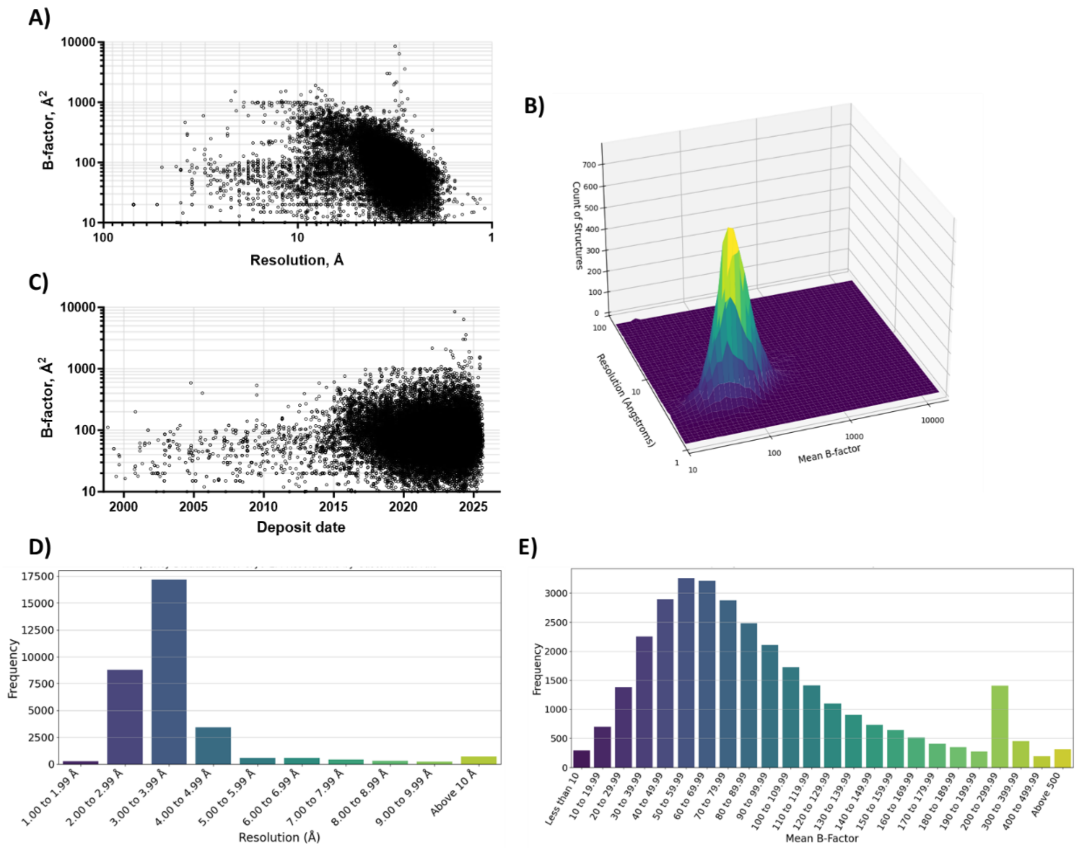

Figure S3. Distribution of B-factors for cryoEM data. A) Mean B-factor vs. Resolution for Cryo-EM Structures. **B)** 3D Distribution of mean B-factor vs. resolution for cryo-EM structures. **C)** Mean B-factor vs. deposit date for cryo-EM structures. **D)** Frequency distribution of cryo-EM resolutions. **E)** Frequency distribution of mean B-factors of cryo-EM structures. Data were obtained by querying the cryo-EM structures from the RCSB Protein Data Bank (accessed March 13, 2026; hits corresponding to data deposit between 12/3/1996 and 2/13/2026), downloading the structure files (mmCIF) from 32,431 hits, extracting atomic B-factors present in 31,887 hits, and computing the average B-factor per structure. Graphs were produced with GraphPad Prism 101.2 (panels A and B) and Matplotlib (panel C).
Below is the script run in *Google Colab* (https://colab.research.google.com).

```
# Install packages
!pip install gemmi pandas requests tqdm
import requests
import pandas as pd
import gemmi
from tqdm import tqdm

# -----------------------------
# Step 1: Query cryo-EM entries
# -----------------------------
```

```
query = {
  "query": {
    "type": "terminal",
    "service": "text",
    "parameters": {
      "attribute": "exptl.method",
      "operator": "exact_match",
      "value": "ELECTRON MICROSCOPY"
    }
  },
  "return_type": "entry",
  "request_options": {
      "return_all_hits": True
  }
}
url = "https://search.rcsb.org/rcsbsearch/v2/query"
response = requests.post(url, json=query)
data_json = response.json()
if "result_set" not in data_json:
    raise Exception("Query failed. API response did not contain result_set")
pdb_ids = [x["identifier"] for x in data_json["result_set"]]
print("Total cryoEM structures:", len(pdb_ids))

# -----------------------------
# Step 2: Metadata function
# -----------------------------
def get_metadata(pdb_id):
    url = f"https://data.rcsb.org/rest/v1/core/entry/{pdb_id}"
    r = requests.get(url).json()
    resolution = None
    if "rcsb_entry_info" in r:
        res = r["rcsb_entry_info"].get("resolution_combined")
        if res:
            resolution = res[0]
    method = r["exptl"][0]["method"]
    deposit_date = r["rcsb_accession_info"]["deposit_date"]
    return resolution, method, deposit_date


# -----------------------------
# Step 3: Extract B-factors
# -----------------------------
def get_bfactor(pdb_id):
    cif_url = f"https://files.rcsb.org/download/{pdb_id}.cif"
    r = requests.get(cif_url)
    open("temp.cif","wb").write(r.content)
    structure = gemmi.read_structure("temp.cif")
```

```
    b_vals = []
    for model in structure:
        for chain in model:
            for residue in chain:
                for atom in residue:
                    if atom.b_iso > 0:
                        b_vals.append(atom.b_iso)
    if len(b_vals) == 0:
        return None
    return sum(b_vals)/len(b_vals)

# ----------------------------
# Step 4: Build dataset
# ----------------------------
rows = []
for pdb in tqdm(pdb_ids):
    try:
        res,method,date = get_metadata(pdb)
        bfactor = get_bfactor(pdb)

        rows.append({
            "PDB_ID": pdb,
            "Experimental_Method": method,
            "Resolution": res,
            "Deposit_Date": date,
            "Mean_B_Factor": bfactor
        })
    except:
        continue
df = pd.DataFrame(rows)

# ----------------------------
# Step 5: Save CSV
# ----------------------------
df.to_csv("cryoEM_structures_Bfactor_dataset.csv", index=False)
print("Dataset complete!")

# ----------------------------
# PLOT 3D
# ----------------------------

import pandas as pd
df = pd.read_csv('/content/cryoEM_structures_Bfactor_dataset_ORIGINAL.csv')

# Display the first few rows to confirm it loaded correctly
display(df.head())
print(f"DataFrame loaded with {len(df)} rows and {len(df.columns)} columns.")
```

```
import matplotlib.pyplot as plt
from mpl_toolkits.mplot3d import Axes3D
import numpy as np
import pandas as pd
from matplotlib import ticker

# Ensure 'Resolution' and 'Mean_B_Factor' are numeric and handle potential None values
df_3d = df.dropna(subset=['Resolution', 'Mean_B_Factor']).copy()
df_3d['Resolution'] = pd.to_numeric(df_3d['Resolution'], errors='coerce')
df_3d['Mean_B_Factor'] = pd.to_numeric(df_3d['Mean_B_Factor'], errors='coerce')
df_3d.dropna(subset=['Resolution', 'Mean_B_Factor'], inplace=True)

# Filter for positive values and Mean_B_Factor >= 10 before taking log
df_3d = df_3d[df_3d['Resolution'] > 0]
df_3d = df_3d[df_3d['Mean_B_Factor'] >= 10] # Filter B-factor to start at 10

# Use log scale for binning Resolution and Mean_B_Factor
log_resolution = np.log10(df_3d['Resolution'])
log_b_factor = np.log10(df_3d['Mean_B_Factor'])

# Create 2D histogram
hist, xedges, yedges = np.histogram2d(log_b_factor, log_resolution, bins=50)

# Create meshgrid for the surface plot
X, Y = np.meshgrid(xedges[:-1], yedges[:-1])
Z = hist.T  # Transpose the histogram for correct orientation
fig = plt.figure(figsize=(16, 10))
ax = fig.add_subplot(111, projection='3d')

# Plot the surface
ax.plot_surface(X, Y, Z, cmap='viridis', edgecolor='none')

# Set labels and title with appropriate context
ax.set_xlabel('Mean B-factor', fontsize=12)
ax.set_ylabel('Resolution (Angstroms)', fontsize=12)
ax.set_zlabel('Count of Structures', fontsize=12)
ax.set_title('3D Distribution of Mean B-factor vs. Resolution for Cryo-EM Structures', fontsize=14)

# Manually set logarithmic ticks with original value labels
# For X-axis (Mean B-factor)
min_log_b = np.min(log_b_factor)
max_log_b = np.max(log_b_factor)
b_factor_ticks_original = np.array([10**i for i in range(int(np.floor(min_log_b)), int(np.ceil(max_log_b))
+ 1) if 10**i >= 10])
b_factor_ticks_log = np.log10(b_factor_ticks_original)
ax.set_xticks(b_factor_ticks_log)
ax.set_xticklabels([f'{int(tick)}' for tick in b_factor_ticks_original])
```

```
ax.set_xlim(min_log_b, max_log_b) # Set limits based on filtered log data

# For Y-axis (Resolution) - Updated to 1 to 100
min_log_res = np.log10(.9) # Start at 1 Angstrom
max_log_res = np.log10(100) # End at 100 Angstroms
resolution_ticks_original = np.array([1, 10, 100]) # Specific ticks for 1, 10, 100
resolution_ticks_log = np.log10(resolution_ticks_original)
ax.set_yticks(resolution_ticks_log)
ax.set_yticklabels([f'{int(tick)}' for tick in resolution_ticks_original]) # Display as integers
ax.set_ylim(min_log_res, max_log_res) # Set limits based on log data

# Add specific view angle for better visualization
ax.view_init(elev=30, azim=-110) # Adjust elevation and azimuth for best view
plt.show()


# -----------------------------
# PLOT Resolution distribution
# -----------------------------
import pandas as pd
import matplotlib.pyplot as plt
import seaborn as sns

# Load the dataset (assuming it's already loaded or this cell is re-executed)
df = pd.read_csv('/content/cryoEM_structures_Bfactor_dataset_ORIGINAL.csv')

# Drop rows where 'Resolution' is NaN
df_filtered = df.dropna(subset=['Resolution']).copy()

# Define custom bins and labels
bins = [1.0, 2.0, 3.0, 4.0, 5.0, 6.0, 7.0, 8.0, 9.0, 10.0, float('inf')]
labels = [
   '1.00 to 1.99 Å',
   '2.00 to 2.99 Å',
   '3.00 to 3.99 Å',
   '4.00 to 4.99 Å',
   '5.00 to 5.99 Å',
   '6.00 to 6.99 Å',
   '7.00 to 7.99 Å',
   '8.00 to 8.99 Å',
   '9.00 to 9.99 Å',
   'Above 10 Å'
]

# Categorize 'Resolution' into the defined bins
df_filtered['Resolution_Category'] = pd.cut(
   df_filtered['Resolution'],
```

```
    bins=bins,
    labels=labels,
    right=False, # Includes the left edge, excludes the right edge
    include_lowest=True # Include the lowest bin edge
)

# Count the occurrences in each category and sort them
resolution_counts = df_filtered['Resolution_Category'].value_counts().sort_index()

# Create the bar plot
plt.figure(figsize=(12, 7))
sns.barplot(x=resolution_counts.index, y=resolution_counts.values, palette='viridis')
plt.title('Frequency Distribution of Cryo-EM Resolutions by Custom Intervals', fontsize=16)
plt.xlabel('Resolution (Å)', fontsize=20)
plt.ylabel('Frequency', fontsize=20)
plt.xticks(rotation=45, ha='right', fontsize=18)
plt.yticks(fontsize=18)
plt.grid(axis='y', alpha=0.75)
plt.tight_layout()
plt.show()


# -----------------------------
# PLOT B-factors distribution
# -----------------------------

import pandas as pd
import matplotlib.pyplot as plt
import seaborn as sns

# Ensure df is loaded. If not, load it from the CSV.
# Assuming df is already available from previous steps. If not, uncomment the line below:
# df = pd.read_csv('/content/cryoEM_structures_Bfactor_dataset_ORIGINAL.csv')

# Drop rows where 'Mean_B_Factor' is NaN
df_filtered_bfactor = df.dropna(subset=['Mean_B_Factor']).copy()

# Define custom bins for B-factor
bins_bfactor = [
    float('-inf'), # For 'Less than 10'
    10,
    20, 30, 40, 50, 60, 70, 80, 90, 100, 110, 120, 130, 140, 150, 160, 170, 180, 190, 200, # Intervals of 10
up to 200
    300, 400, 500, # Intervals of 100 up to 500
    float('inf') # For 'Above 500'
]
```

```
# Define custom labels for B-factor bins
labels_bfactor = [
    'Less than 10',
    '10 to 19.99',
    '20 to 29.99',
    '30 to 39.99',
    '40 to 49.99',
    '50 to 59.99',
    '60 to 69.99',
    '70 to 79.99',
    '80 to 89.99',
    '90 to 99.99',
    '100 to 109.99',
    '110 to 119.99',
    '120 to 129.99',
    '130 to 139.99',
    '140 to 149.99',
    '150 to 159.99',
    '160 to 169.99',
    '170 to 179.99',
    '180 to 189.99',
    '190 to 199.99',
    '200 to 299.99',
    '300 to 399.99',
    '400 to 499.99',
    'Above 500'
]

# Categorize 'Mean_B_Factor' into the defined bins
df_filtered_bfactor['B_Factor_Category'] = pd.cut(
    df_filtered_bfactor['Mean_B_Factor'],
    bins=bins_bfactor,
    labels=labels_bfactor,
    right=False, # Includes the left edge, excludes the right edge
    include_lowest=True # Include the lowest bin edge
)

# Count the occurrences in each category and sort them
bfactor_counts = df_filtered_bfactor['B_Factor_Category'].value_counts().sort_index()

# Create the bar plot
plt.figure(figsize=(15, 8))
sns.barplot(x=bfactor_counts.index, y=bfactor_counts.values, palette='viridis')
plt.title('Frequency Distribution of Mean B-Factors by Custom Intervals', fontsize=16)
plt.xlabel('Mean B-Factor', fontsize=20)
plt.ylabel('Frequency', fontsize=20)
plt.xticks(rotation=60, ha='right', fontsize=18)
```

```
plt.yticks(fontsize=18)
plt.grid(axis='y', alpha=0.75)
plt.tight_layout()
plt.show()


# -----------------------------
# END
# -----------------------------
```
